# Revisiting Compassion and Job Performance: A Constructive Study in South Korean Public Art Institutions

**DOI:** 10.3390/bs14100963

**Published:** 2024-10-17

**Authors:** Pilyun Ahn, Sung-Hoon Ko, Yongjun Choi

**Affiliations:** 1 Department of Three Dimensional Art, College of Arts, Kyonggi University, Suwon 16227, Republic of Korea; pyahn3@naver.com; 2 Graduate School of Education, Kyonggi University, Suwon 16227, Republic of Korea; 3 College of Business Administration, Hongik University, Seoul 04066, Republic of Korea; yongjun.choi@hongik.ac.kr

**Keywords:** compassion, positive work-related identity, positive psychological capital, job performance, art sector, cultural workers, public institutions

## Abstract

This study empirically examines how employee compassion relates to job performance. Specifically, this study is a constructive replication and expansion of a previous study on the relationship between compassion and job performance using multiple sources of measurement. It investigates unexplored pathways within the public art sector in South Korea. Focusing on the mediating roles of positive work-related identity (PWRI) and positive psychological capital, we collected data from public art institutions in Korea, including galleries and museums, using a survey method. We tested the hypotheses using structural equation modeling and the PROCESS bootstrapping method. Our findings demonstrate a positive association between compassion and job performance, serially mediated by PWRI and positive psychological capital. Theoretically, by constructively replicating and expanding the previous findings, our study contributes to a robust understanding of how compassion could enhance employee performance. Practically, this study reinforces the value of fostering compassion and positive psychological resources to improve job performance, particularly within the public art sector.

## 1. Introduction

Lilius et al. [1] define compassion as “a multi-dimensional process in which three elements of compassion form a tri-partite concept: noticing another person’s suffering, empathically feeling that person’s pain, and acting in a manner intended to ease the suffering” (pp. 194–195). To date, empirical studies have focused on the relationship between compassion experienced by employees and their performance [1,2,3]. When examined from the perspectives of cognitive consciousness and affective empathy, compassion signifies solidarity with others who are in difficulty and suffering and encompasses care for others [4,5,6]. Examples of suffering may include illness or mental trauma, the death of a loved one, and work-related stress that can arise in the workplace [7]. Compassion, which responds to such suffering through action, is a source of care and healing for other employees who are in difficulty [4,8].

This study explores how the compassion experienced by employees in the Korean art sector (i.e., cultural workers) helps alleviate their suffering while improving their job performance. Furthermore, it explores whether the exchange of compassionate care among cultural workers transforms their work-related identity and enhances their positive psychological capital. Public art institutions were selected for this study for the following two primary reasons: First, cultural workers have undergone rigorous apprenticeship training in fields such as art, music, and sports, leading to a predominantly vertical organizational culture [9]. Hence, this present study aims to examine how compassion among cultural workers, who may be distanced from such sentiments due to their training, influences job performance [10]. Second, cultural workers, primarily art majors, are expected to possess high emotional intelligence and greater empathy [11]. Thus, we chose this sample to examine the positive emotions that acts of compassion may evoke in a context characterized by employees with relatively high levels of emotional intelligence [12].

Lilius et al. [1] were the first to investigate the relationship between compassion and performance variance and developed a scale for measuring compassion. Their study represents a groundbreaking contribution to organizational behavior. In addition, Ko and Choi [3] empirically examined the relationship between compassion and job performance, describing the relationship between mediating variables and performance variables based on the self-consistency theory [13,14,15]. Similarly, Hur et al. [16] also empirically investigated the relationship between compassion, job performance, and creativity, describing the relationship between compassion and job performance based on the amplifying and buffering effects identified in Cameron et al.’s [17] studies. Additionally, the study by Rhee et al. [18] elucidates the mechanisms between compassion and performance variables based on the job demands–resources (JD–R) model.

Previous studies that empirically examined the mechanisms through which compassion relates to performance variables investigated several mediating factors, including positive emotions [1], positive psychological capital [3,19], positive work-related identity (PWRI) [3], and collective self-esteem [3]. This study focuses on the mediating roles of PWRI and positive psychological capital in the relationship between compassion and job performance. While Ko and Choi [3] investigated these variables, they did not examine the serial mediation of PWRI and positive psychological capital together. The current study addresses this gap by replicating and extending Ko and Choi’s [3] findings, analyzing how the compassion experienced by cultural workers influences job performance through the serial mediating effects of PWRI and positive psychological capital. The primary objective is to enhance our understanding of compassion’s role in the workplace.

First, we constructively replicate and extend Ko and Choi’s [3] study. Specifically, while Ko and Choi [3] examined the roles of PWRI and psychological capital separately in the positive relationship between compassion and job performance, our study simultaneously investigates the serial mediating roles of PWRI and psychological capital in a research model. This approach contributes to a more comprehensive understanding of the mechanisms linking compassion to job performance. Second, while Ko and Choi [3] focused on full-time employees in large-sized companies, our study specifically targets cultural workers in public art institutions. This sample is likely to require compassionate care more than general office employees, thereby enhancing the relevance of our findings within this particular context. Doing so increases the generalizability of Ko and Choi’s [3] findings. Third, our study reinforces the findings of Ko and Choi [3] through methodological advancement. Specifically, whereas Ko and Choi [3] relied on self-reported measures of job performance, which may lead to biases related to leniency or social desirability, our study uses a supervisor’s rating to assess the job performance of cultural workers. Using multisource data, we provide a more robust understanding of how compassion influences job performance.

## 2. Hypotheses Development

### 2.1. Compassion and Positive Work-Related Identity (PWRI)

Suffering refers to the pain and wounds that cause existential hardship and misery. Examples of suffering include personal illnesses and injuries, psychological injuries, physical pain, mental stress, and emotional exhaustion that may occur at work [20]. Thus, personal compassion is a source of caring and resilience, hope for the future, and optimism for others experiencing suffering within the corporate organization [4,8]. Lilius et al. [1] investigated how the compassion employees give and receive impacts their positive emotions and emotional commitment. They found that employees who gave or received compassion in response to suffering are more likely to experience positive emotions such as pride, joy, inspiration, and comfort. Likewise, past studies also provide support that compassion generated within an organization induces positive emotions [21,22,23].

The social identity theory [24,25] posits that individuals form their identities through interactions in various social contexts and by categorizing themselves into groups, distinguishing themselves from others [24,25,26,27]. From the social identity theory perspective, the employees who experience compassion within an organization are more likely to increase their intrinsic motivation to enhance organizational identity when they perceive themselves as members of a group with positive distinctiveness. Social categorization often leads group members to favor their in-group over the out-group [21,26]. As a result, compassionate behaviors within the organization encourage members to view themselves and their organization more positively, prompting them to engage in identity-enhancing behaviors. Dutton et al.’s [21] four types of PWRI (i.e., virtue, evaluative, developmental, and structural perspectives) derive from the social identity theory.

Previous research has shown that compassion can influence employee attitudes and identities by shaping their sensemaking of the organization to which they belong and the colleagues with whom they work [1]. In other words, employee perceptions of the organization’s acts of compassion (i.e., giving, receiving, or witnessing compassion) affect their subsequent identities, attitudes, and behaviors [1]. Additionally, narratives within the organization convey compassion, which changes employee self-concepts and the perceptions of the organization.

The employees who experience compassion tend to talk about how their organization or colleagues genuinely treat them. As employees share these compassion narratives, they develop positive perceptions of their organization, identifying it as a care-providing system [22] and a source of social support and healing [28]. According to Lilius et al. [1], the employees who experience compassion are more likely to perceive it positively and experience positive emotions, as they feel their organization genuinely cares for them. This perception leads them to see themselves as competent, capable, accepted, and valued by others. According to the social identity theory, employees develop a positive work-related identity when they experience positive emotions toward the organization through compassionate acts. This process indicates that individuals shape their identities through interactions with others [24,25]. Therefore, we suggest that the employees who experience compassion at work are more likely to develop a PWRI, allowing us to hypothesize as follows:

**Hypothesis H1.**  *Compassion positively relates to positive work-related identity*.

### 2.2. Positive Work-Related Identity (PWRI) and Positive Psychological Capital

Employee work-related identity becomes more positive when associated with virtuous qualities, compassionate attributes, or character strengths that distinguish them positively from others [21]. Therefore, when employees experience compassion within the organization, their identity as employees forms more positively. As a result, employees with a PWRI desire to maintain high levels of self-efficacy and self-esteem, which are sub-dimensions of positive psychological capital [2]. Additionally, past studies show that PWRI has a positive relationship with hope and optimism, sub-dimensions of positive psychological capital [29,30].

The evaluative perspective of PWRI assumes that employee work-related identity is positive when favorably evaluated by individuals or others [21]. The extent to which they desire to be recognized for their value or evaluated as important, internally or externally, motivates employees to adjust their attitudes and behaviors toward the organization [24,25,26]. When employees engage in compassionate behaviors and see themselves as part of a collective group with a positive image and reputation, they become more motivated to enhance their identities [26]. Therefore, based on the evaluative perspective of PWRI, the formation of a PWRI by employees in the organization to receive favorable evaluations from the organization and others will positively impact hope and optimism regarding the future, which are sub-dimensions of positive psychological capital.

On the other hand, the employees who form a PWRI through compassion experience resilience, which is a sub-dimension of positive psychological capital. Therefore, we predict a positive relationship between PWRI and positive psychological capital [2,21]. From the developmental perspective of PWRI, when individuals within the organization perceive their identity as advancing to a higher stage, better aligning internal or external standards, they experience psychological resilience, a sub-dimension of positive psychological capital.

In other words, the employees who experience compassionate actions are likely to develop a more positive work-related identity, thereby gaining resilience from the developmental perspective of PWRI [21]. As employees experience and engage in compassionate behaviors, they undergo mental maturity, moving from indifference to helping others, enhancing their resilience, a sub-dimension of positive psychological capital. Therefore, based on previous research on PWRI and positive psychological capital, we predict that the employees who form a PWRI will ultimately develop positive psychological capital.

**Hypothesis H2.**  *Positive work-related identity positively relates to positive psychological capital*.

### 2.3. Positive Psychological Capital and Job Performance

Scholars have shown that social capital, along with trust, cooperation, and value sharing among employees, reduces organizational cynicism and turnover intention while enhancing job performance and organizational commitment, thereby contributing to improved organizational performance [31]. In this regard, Luthans [32] argued for developing personal positive organizational behavior to improve organizational performance and established the definition of positive psychological capital. Positive organizational behavior emphasizes psychological values through the concept of psychological capital based on psychology and believed to contribute more to production than traditional capital [32,33]. Positive psychological capital awakens an individual’s existing maximum potential beyond human capital (i.e., knowledge, skills, and experiences) and transcends social capital (i.e., individual networks or relationships) [34]. Positive psychological capital consists of four independent components: self-efficacy, optimism, hope, and resilience [35].

Positive psychological capital is vital because it can induce positive changes in an employee’s attitudes, behaviors, and performance and promote positive organizational changes. The positive relationship between positive psychological capital and job performance derives from Fredrickson’s [36] broaden and build theory of positive emotions. An individual’s positive emotions expand thoughts and actions, forming continuous personal resources ranging from physical and intellectual resources to social and psychological resources. The increase in these resources leads to the upward development and growth of employees, ultimately positively impacting job performance. In other words, according to Fredrickson [36], positive psychological capital enables employees to experience a virtuous cycle of positive emotions, which positively influences job performance.

Prior studies have revealed that self-efficacy, a sub-dimension of positive psychological capital, significantly impacts job performance depending on the effort an employee invests in a specific task for which they are responsible [37]. Studies have also revealed that hope and optimism as sub-dimensions of positive psychological capital are the driving forces to overcome difficulties like job stress and emotional exhaustion at work, thus helping improve job performance [38,39]. Research empirically supports self-efficacy as a sub-dimension of positive psychological capital as positively affecting various work-related performance variables [40]. In addition, it is not difficult to find research findings that show a positive effect of hope on outcome variables such as job performance, job satisfaction, and job attitude [39]. The literature also reveals that employees with positive optimism show higher job performance than those without [41]. Therefore, we hypothesize the following:

**Hypothesis H3.**  *Positive psychological capital positively relates to job performance*.

### 2.4. Compassion and Job Performance

Recent studies have shown that compassion at work has a positive causal relationship with employees and organizational function [1,42,43,44,45]. Just as compassion is an empathetic response to the suffering of others [45], experiencing compassion from other employees (i.e., giving and receiving temporal, mental, and material support) plays a crucial role in employee resilience in the workplace environment where suffering coexists [44]. In addition, experiencing compassion within an organization induces positive emotions such as pride, gratitude, and inspiration and reinforces emotional commitment [1]. Compassion helps employees achieve a more balanced work life and induces important organizational performance [44]. Also, employees experiencing compassion tend to have more supportive behavior [46,47].

Dutton et al. [42] argue that when employees experience compassion within an organization, it develops the organization’s propensity for cooperation by generating relational resources, strengthening shared values, and cultivating important interpersonal skills. Overall, previous research suggests that experiencing compassion in the organization directly benefits suffering employees by alleviating pain, anxiety, and sadness. It also indirectly benefits other employees by enhancing emotional commitment, positive emotions, and job performance and reducing turnover intention [3,16]. 

Alessandri et al. [48] suggested that the experience of providing compassion can enhance the feelings of self-efficacy as a capable contributor. Employees with high self-efficacy likely effectively and successfully use and generate resources within the organization to perform the required tasks competently within a specified timeframe [49]. Bandura [50] found that individuals with strong self-efficacy are more capable of resolving painful and threatening situations than those with weak self-efficacy. Additionally, previous research on nurses revealed that those who perceive themselves as having high self-efficacy can successfully resolve conflicts with patients or cope with job-related demands [51]. In other words, the individuals who provide compassion to others may enhance their self-perceived self-efficacy, thereby improving job performance. Providing compassion can elicit positive emotions, positively affecting the provider and the job performance of the employees receiving compassion [1,48].

According to the affective event theory, providing or receiving compassion can be a significant affective event within the organization, and employees who receive compassionate acts experience positive emotions [52,53]. When employees provide or receive compassion, their relationships with others intensify, leading to positive emotions [54]. Employees exhibiting high positive emotional states show improved job performance compared to those with lower positive emotional states [55]. Therefore, we hypothesize the following:

**Hypothesis H4.**  *Compassion positively relates to job performance*.

### 2.5. Serial Multiple Mediation of Positive Work-Related Identity and Positive Psychological Capital

Employees experience compassion as an emotional response to the suffering of others, leading them to feel positive emotions about their work, thus fostering a PWRI [1,21,22,56]. Experiencing compassion within an organization evokes positive feelings about personal and social identities among employees, thereby contributing to a positive mental state [1]. Based on the social identity theory [24,25], the employees who experience compassion within an organization are likely to develop a PWRI. That is, when employees exchange acts of compassion, they feel positive emotions toward their organization and subsequently form a positive identity, ultimately leading to a positive perception of their work-related identity.

The PWRI formed through compassion consists of four perspectives: virtue, evaluative, developmental, and structural [21]. From the evaluative perspective of PWRI, employees enhance their hope and optimism, which are sub-dimensions of positive psychological capital, by receiving favorable evaluations from other employees. Moreover, from the developmental perspective of PWRI, employees recognized as virtuous by others experience resilience, another sub-dimension of positive psychological capital [21]. Additionally, from the virtue perspective of PWRI, employees improve their physical health and happiness, enhancing self-efficacy, a sub-dimension of positive psychological capital, while experiencing buffering effects and resilience [57]. Therefore, the employees who exchange compassion develop the four perspectives of PWRI (virtue, evaluative, developmental, and structural), leading to an increase in positive psychological capital.

Previous research shows that self-efficacy, a sub-dimension of positive psychological capital, significantly impacts job performance depending on employees’ efforts [37]. Additionally, studies indicate that hope and optimism are motivators for overcoming challenges such as job stress and emotional exhaustion, thereby aiding in enhancing job performance [38,39]. In other words, empirical evidence supports that self-efficacy positively influences various job-related performance variables [40,58], and research shows that hope positively affects performance variables such as job performance, job satisfaction, and job attitudes [39]. Other studies have also revealed that employees with positive optimism demonstrate higher job performance than those without [41].

According to the broaden and build theory [36], positive psychological capital generates positive emotions, ultimately enhancing job performance. In other words, the PWRI formed through acts of compassion induces positive emotions, which expand employees’ thoughts and actions, leading to improved job performance [36]. Hence, we predict a dual mediation hypothesis of PWRI and positive psychological capital among employees.

**Hypothesis H5.**  *Positive work-related identity and positive psychological capital serially mediate the positive relationship between compassion and job performance*.

**Figure 1:**
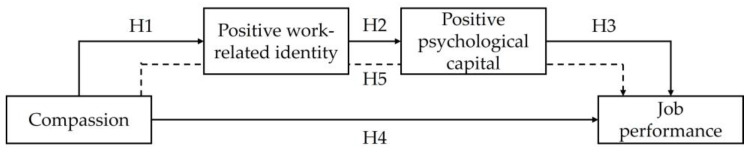
Research model. The dotted line denotes the serial mediation hypothesis.

Figure 1 presents this study’s research model.

## 3. Method

### 3.1. Study Participants and Procedure 

Data were collected from a panel of cultural workers employed in public art institutions in South Korea. Specifically, we utilized a cross-sectional design with an online survey to collect the data. The data collection period spanned from early July to mid-July 2024. Accordingly, this study used a sample of 409 individuals, all cultural workers specializing in arts and employed at public art institutions in South Korea, including galleries and museums. The final sample comprised 199 males (48.7%) and 210 females (51.5%). The age distribution of the sample included 67 individuals in their 20s (16.4%), 152 in their 30s (37.2%), 119 in their 40s (29.1%), 64 in their 50s (15.6%), and 7 in their 60s (1.7%). The main occupations of the employees working in public art institutions were curators and art-related professionals, totaling 266 individuals (65%), followed by 70 (17.1%) who were art majors working in administrative roles. Art majors responsible for public relations and marketing accounted for 50 individuals (12.2%), while craft designers numbered 18 (4.4%), and visual merchandisers totaled 5 (1.2%). The duration of employment was as follows: 156 individuals (38.1%) had worked for 1 to 4 years, 165 (40.3%) for 5 to 9 years, 58 (14.2%) for 10 to 14 years, 27 (6.6%) for 15 to 19 years, and 3 (0.7%) for over 20 years. Our samples’ educational background comprised 155 individuals (37.9%) who graduated from a two-year college, 200 (48.9%) from a four-year university, 51 (12.5%) were enrolled in or graduated from a master’s program, and 3 (0.7%) were enrolled in or graduated from a doctoral program.

### 3.2. Measures

Survey items are presented in Appendix A.

#### 3.2.1. Compassion

This study measured compassion using three items from Lilius et al. [1]. Lilius et al. [1] defined compassion as “noticing another person’s suffering, empathically feeling that person’s pain, and acting in a manner that intended to ease the suffering” (pp. 194–195), which aligns with the purpose of our study. Prior empirical research using the compassion variable, such as Moon et al. [2], Hur et al. [16], and Rhee et al. [18], also utilized the compassion scale developed by Lilius et al. [1], providing empirical evidence of the scale’s reliability and validity. The compassion items used in this study are as follows: “While working in an art-related public institution, I provided and received emotional, temporal, and material support when my colleagues were in suffering”, “While working in an art-related public institution, I provided and received emotional, temporal, and material support when my subordinates were in suffering”, and “While working in an art-related public institution, I provided and received emotional, temporal, and material support when my supervisor was in suffering”. We assessed the measurement items on a five-point Likert scale (1 = strongly disagree to 5 = strongly agree), and the reliability coefficient, Cronbach’s alpha, was 0.873.

#### 3.2.2. Positive Work-Related Identity (PWRI)

We measured PWRI using four items from Bednar et al. [29] derived from the discussion by Dutton et al. [21]. To date, empirical research on PWRI is scarce, and there is a lack of consensus among researchers regarding what constitutes PWRI. Dutton et al. [21] defined PWRI from four perspectives: virtue, evaluative, developmental, and structural. Therefore, we concluded that Bednar et al. [29] could capture a comprehensive picture of PWRI. In addition, Moon et al. [2] and Hur et al. [16] also used the same measurement, providing adequate evidence of its reliability and validity. Example items include “As a member of this public institution, I am a person of virtuous character” and “As a member of this public institution, I exemplify virtuous qualities”. We rated each item on a five-point Likert scale (1 = strongly disagree to 5 = strongly agree), and the reliability coefficient, Cronbach’s alpha, was 0.916.

#### 3.2.3. Positive Psychological Capital

We used Youssef and Luthans’ [39] scale to measure positive psychological capital. Specifically, we used 24 measurement items employed in Luthans et al. [35] that drew on Youssef and Luthans’ [39] study. The items included “I can come up with many ways to overcome difficulties when faced with hardship at work”, “I can overcome and recover from frustration and despair at work”, and “I do not feel much difficulty”.

We rated each item on a five-point Likert scale (1 = strongly disagree to 5 = strongly agree), and the Cronbach’s alphas for the sub-dimensions of positive psychological capital were as follows: hope α = 0.880, resilience α = 0.891, self-efficacy α = 0.893, and optimism α = 0.894 in Luthans and Youssef [30]. In this study, Cronbach’s alpha for the construct of positive psychological capital was 0.921.

#### 3.2.4. Job Performance

This study measured job performance by modifying the four items used by Gong et al. [59]. Supervisors at the team leader level and above within public art institutions rated their subordinates’ job performance. Sample items include “My subordinate makes significant contributions to the overall performance of our department”, “My subordinate is one of the best employees in our department”, and “My subordinate always completes job assignments on time”. The supervisors provided their ratings using a five-point Likert scale (1 = strongly disagree to 5 = strongly agree), and the reliability coefficient, Cronbach’s alpha, was 0.880.

#### 3.2.5. Control Variables

In this study, a correlation analysis showed that the working period among the demographic variables had a significant relationship with the latent variables. After controlling for the working period, we tested the hypotheses using structural equation modeling. We also controlled for the mixed effect that the individual’s propensity characteristics can have on the main variables of this study.

## 4. Results

### 4.1. Confirmatory Factor Analysis (CFA)

This study verified discriminant validity by calculating the average variance extraction (AVE), with reliability verified by calculating Cronbach’s alpha values. The Cronbach’s alphas for each latent variable scale satisfied the traditional standard, with all the values exceeding 0.7. The AVE value also satisfied the traditional standards, showing a value exceeding 0.6.

The results of the confirmatory factor analysis (CFA) went as follows: χ^2^(450) = 868.307; *p* < 0.001. The latent variables’ measurement items employed in this study satisfied the traditional criteria; Table 1 supports our judgment that all the values from the CFA are satisfactory [60].

### 4.2. Correlation Analysis

The fitness values for the measures used in each variable were satisfactory to the traditional standards, and we tested the questionnaire for convergent validity to determine each variable’s representativeness [61]. In addition, to verify whether each item correlated with the variables in two or more research models, we assessed discriminant validity.

This study conducted Pearson correlation analysis and regression analysis to examine multicollinearity before interpreting the results of structural equation modeling (SEM) to test the hypotheses. After conducting a confirmatory factor analysis to verify the suitability of the research model, we selected 5 questions for compassion, 4 for PWRI, 21 for positive psychological capital, and 5 for job performance. We then performed a correlation analysis, finding that the correlation values between the latent variables ranged from 0.209 to 0.478 (*p* < 0.01). Table 2 presents the correlation analysis results. 

### 4.3. Hypotheses Testing

We used the Amos 27.0 program to verify the hypotheses with SEM and demonstrate the mechanism of competition and job performance experienced by cultural workers within the organization. After conducting the confirmatory factor analysis, we removed the measurement items with high correlations or very low factor loads (λ) (i.e., PsyCapital14, PsyCapital17, and PsyCapital19). For SEM testing, we selected 3 items to measure compassion, 4 to measure PWRI, and 21 to measure positive psychological capital. Table 3 shows that the SEM results support Hypotheses H1–H4.

### 4.4. The Double Mediation Effect of Positive Work-Related Identity and Positive Psychological Capital in the Relationship Between Compassion and Job Performance

This study employed the PROCESS bootstrapping method to test the double mediation effect [62]. Bootstrapping is useful in judging whether the indirect effect is statistically significant (CI 95% = LLCI value, ULCI value). As per Table 4, the indirect effect between compassion and job performance was between LLCI 95% = 0.051 and ULCI 95% = 0.146, confirming the double mediation effect’s significance and thereby supporting Hypothesis H5. The results of verifying the dual mediation effects indicate that the compassion experienced by cultural workers within the organization significantly influences job performance by fostering positive psychological capital through PWRI.

### 4.5. Common Method Bias

This study analyzed data gathered through self-reports. As a result, common method bias may affect the validity of the research results [63]. Scholars commonly use Harman’s [64] single-factor test to verify the possibility of common method bias. However, in this study, we address common method bias by applying a latent variable factor control method that does not involve direct measurement.

The latent variable factor control method utilizes the significance of the χ^2^ change according to the change in the degree of freedom, the change in the fit of the research model, the estimate of the path coefficient, and the significance evaluation [63,65]. In other words, if the amount of change in χ^2^ according to the change in the degree of freedom is significant by comparing before and after the control of latent method factors, there is a common method bias.

Because we used self-reported data for hypothesis testing, we acknowledge that our results might be biased due to common method variance. Hence, we conducted the latent variable approach [63]. The results are in Table 5. Before controlling for the latent variables, the measurement model fit indices were χ^2^(450) = 863.307, *p* < 0.001, CFI = 0.936, TLI = 0.925, IFI = 0.937, RMSEA = 0.037, and RMR = 0.030. After controlling for the latent variables, the model fit indices were χ^2^(417) = 648.737, *p* < 0.001, CFI = 0.965, TLI = 0.955, IFI = 0.965, RMSEA = 0.037, and RMR = 0.021. The χ^2^ difference (df = 33) was not statistically significant (*p* > 0.05). Thus, we concluded that common method variance is not a serious issue in our study’s model.

Therefore, the analysis for hypothesis testing used a model before controlling for latent method factors [65]. In addition, as per Table 6, the absolute value of the λ-CMV values, which is the difference between the λ value before and after control, did not exceed 0.2; thus, the probability of common method bias among the latent variables used in this research model is low [63,66].

## 5. Discussion

### 5.1. Theoretical Implications

This study elucidates the mechanisms of how compassionate behaviors among cultural workers employed in public art institutions relate to their job performance, along with the serial mediating roles of positive work-related identity (PWRI) and positive psychological capital. Our findings provide the following theoretical contributions.

First, this study provides empirical evidence elucidating how compassion exchanged among employees transforms job performance, along with the roles of PWRI and positive psychological capital. Ko and Choi [3] investigated the mediating roles of PWRI and psychological capital in the positive relationship between compassion and job performance. However, the researchers did not simultaneously consider PWRI and psychological capital in a research model. Hence, by empirically testing the serial mediation effect of PWRI and psychological capital, our findings offer a more comprehensive understanding of how compassion influences job performance. Moreover, this study addresses one of the key limitations of Ko and Choi’s [3] study, which relied on self-reported measures of job performance, potentially introducing biases such as leniency or social desirability.

Similarly, it is noteworthy that most previous studies examining the role of compassion in organizational settings have also primarily relied on self-reported measures of performance-related variables (e.g., Hur et al. [16]; Moon et al. [2]). On the other hand, our study employed supervisory ratings to assess employee performance, thereby mitigating common method variance. Using multisource data in this current study enhanced the methodological rigor, providing a more solid understanding of how compassion influences job performance. It reinforces the findings of Ko and Choi [3] and contributes to a deeper theoretical and empirical understanding of the roles of compassion in organizational contexts.

Second, this study constructively replicated Ko and Choi’s [3] study by targeting cultural workers in public art institutions. However, unlike Ko and Choi [3], who focused on general full-time employees in large corporations, our study examined individuals whose roles may require more compassionate care due to their unique emotional and social challenges at work. By exploring compassion within this particular context, our findings enhance their relevance and contribute to the broader generalizability of Ko and Choi’s [3] work. This focus on diverse employment settings underscores the essential role of compassion in promoting positive outcomes across various organizational environments.

Third, this study contributes to the theoretical understanding of compassion’s role within organizations by building on and extending the existing research. Specifically, regarding the roles of PWRI in explaining the effect of compassion at work, while past studies mainly relied on the social identity theory (e.g., Hur et al. [16]; Moon et al. [2]), the present study differentiates itself by applying the social identity theory and the broaden and build theory simultaneously. This approach offers a more insightful perspective on the mechanisms connecting compassion to performance outcomes, thereby enriching the theoretical foundation for understanding the roles of compassion in explaining performance variables in organizational settings.

### 5.2. Practical Implications

First, this study emphasizes the significant impact of positive work-related identity (PWRI) and positive psychological capital, formed through the compassion experienced by cultural workers in public art institutions, on improving job performance. These findings hold practical significance in promoting job performance enhancement through the core competencies required of knowledge workers in the twenty-first century. As Professor Peter Drucker noted, organizations in modern society require knowledge workers to utilize their knowledge actively and collaborate across the company. Therefore, research indicates that compassionate behavior among employees within public institutions can enhance job performance and improve knowledge workers’ organizational capabilities. Additionally, as modern organizations require interdisciplinary and creative talents with core competencies, compassion, which serves as a cause for improving job performance and creativity [3,67], can act as a catalyst for long-term human resource development in companies.

Second, compassion manifests as deep caring behavior among employees, contributing to creating a compassionate and positive organizational culture. Frequent employee turnover can lead to significant cost reductions and energy consumption in recruitment for the organization. Therefore, fostering a compassionate and positive organizational culture within public art institutions will reduce turnover intentions in the long term, securing excellent talent and reducing costs. Specifically, to cultivate a compassionate work environment in public art institutions, it is vital for practitioners to adopt systematic and strategic approaches that integrate a compassionate climate into the organizational goals and strategies. Our findings place emphasis on the practical significance of motivating cultural workers to engage in regular compassion reflection and to promote the use of compassionate language as a fundamental element in fostering a compassionate organizational culture.

Third, compassion, as a response to the suffering of others, fosters a perspective that views others with subjectivity rather than objectivity. For the art majors who have undergone apprenticeship-style art education and work in public institutions, there is a greater need for caring behavior and a strong desire for a horizontal organizational culture. Thus, this study anticipates that the compassionate behavior of art-major employees working in public art institutions will provide a subjective perspective of others at the individual level and contribute to forming a horizontal organizational culture.

### 5.3. Limitations and Future Direction

First, this study’s limitation is that we conducted a cross-sectional study representing only one period. Employees, especially cultural workers, need time after experiencing compassion to form a positive work-related identity and improve job performance. For the emotions of compassion to lead to outcomes, there must be a temporal flow; thus, cross-sectional research has limitations. We suggest that future research include longitudinal studies that measure the impact of compassion on outcome variables over time.

Second, although this study focused on public art institutions, we acknowledge that the nature of the job may moderate the relationship between compassion and job performance. Previous research has not thoroughly investigated this moderating effect (e.g., Hur et al. [16]), leaving an important avenue for future research. For instance, in jobs characterized by high emotional labor (e.g., healthcare and social services), compassion may improve job performance by fostering stronger interpersonal connections [68]. On the other hand, in jobs with low emotional interaction or highly repetitive tasks (e.g., administrative work), compassion may play a less critical role in enhancing job performance [69]. Future research could contribute to a deeper understanding of the roles of compassion by examining whether its impact on job performance varies according to job characteristics.

Third, we acknowledge that personality traits may serve as moderators in the relationship between psychological capital and job performance, as suggested by the trait activation theory [70]. Future research could investigate the moderating effects of various personality traits, such as positive and negative emotional tendencies [71] and emotional intelligence [72]. Additionally, incorporating motivational orientation, as proposed by Carver and White [73], may further enhance our understanding of these dynamics.

Fourth, the data used as a sample for this study came from the metropolitan areas of Seoul and Gyeonggi Province and cultural workers across the country, which provide a certain level of generalizability of the sample. Future research should approach the generalizability of the sample further through cross-national comparative studies that empirically examine the relationship between compassion and outcome variables experienced by the employees working in domestic and foreign public art institutions.

## Figures and Tables

**Table 1:** Confirmatory factor analysis (CFA).

Construct	Items	λ	SE	CR	Cronbach’s ⍺	AVE	CR
Compassion	Com1	0.718	-	-	0.873	0.639	0.881
	Com2	0.647	0.071	12.855			
	Com3	0.929	0.087	14.407			
Positive Work-related Identity	PWRI1	0.897	-	-	0.916	0.834	0.952
	PWRI2	0.890	0.038	26.156			
	PWRI3	0.765	0.043	19.995			
	PWRI4	0.866	0.039	24.802			
Positive Psychological Capital	PsyCapital1	0.661	-	-	0.921	0.712	0.947
	PsyCapital2	0.708	0.082	12.683			
	PsyCapital3	0.655	0.079	11.838			
	PsyCapital4	0.687	0.083	12.362			
	PsyCapital5	0.648	0.081	11.758			
	PsyCapital6	0.669	0.072	12.057			
	PsyCapital7	0.675	0.075	12.222			
	PsyCapital8	0.654	0.076	11.915			
	PsyCapital9	0.482	0.077	9.052			
	PsyCapital10	0.626	0.077	11.425			
	PsyCapital11	0.662	0.076	12.040			
	PsyCapital12	0.595	0.076	10.912			
	PsyCapital13	0.555	0.080	10.248			
	PsyCapital15	0.521	0.074	9.683			
	PsyCapital16	0.503	0.077	9.348			
	PsyCapital18	0.510	0.081	9.469			
	PsyCapital20	0.544	0.071	10.060			
	PsyCapital21	0.462	0.078	8.646			
	PsyCapital22	0.450	0.077	8.416			
	PsyCapital23	0.602	0.088	11.027			
	PsyCapital24	0.534	0.083	9.964			
Job Performance	JP1	0.669	-	-	0.880	0.627	0.981
	JP2	0.717	0.078	14.148			
	JP3	0.726	0.100	12.397			
	JP4	0.706	0.098	12.027			
χ^2^(450) = 868.307 (*p* = 0.000), CFI = 0.936, TLI = 0.925, IFI = 0.937, RMSEA = 0.048, RMR = 0.030							

Note: Com = compassion; PWRI = positive work-related identity; PsyCapital = positive psychological capital; JP = job performance.

**Table 2:** Construct means, standard deviations, and correlations.

	1	2	3	4
1. Compassion	0.799			
2. PWRI	0.209 **	0.913		
3. PsyCapital	0.394 **	0.478 **	0.783	
4. Job Performance	0.286 **	0.302 **	0.328 **	0.791
Mean	3.710	3.560	3.520	3.790
SD	0.650	0.690	0.440	0.600
Max	5.000	5.000	5.000	5.000
Min	1.000	1.000	2.420	2.300

Note: ** *p* < 0.01, the number in the diagonal is the square root of the AVE.

**Table 3:** Path analysis results.

H	Path	B	SE	CR	*p*	Test
H1	Compassion → PWRI	0.222	0.052	4.314	*p* < 0.001	Accept
H2	PWRI → PsyCapital	0.304	0.028	10.992	*p* < 0.001	Accept
H3	PsyCapital → JP	0.441	0.061	7.258	*p* < 0.001	Accept
H4	Compassion → JP	0.138	0.039	3.500	*p* < 0.001	Accept

Note: positive work-related identity = PWRI; positive psychological capital = PsyCapital; job performance = JP.

**Table 4:** Indirect effects for the double mediation effects (PWRI and positive psychological capital).

	Effect	LLCI 95%	ULCI 95%	BootSE
Total indirect effect	0.185	0.115	0.273	0.039
Compassion → PWRI → JP	0.063	0.029	0.107	0.019
Compassion → PsyCapital → JP	0.095	0.051	0.146	0.024
Compassion → PWRI → PsyCapital → JP	0.026	0.010	0.050	0.010

Note: PWRI = positive work-related identity; PsyCapital = positive psychological capital; JP = job performance.

**Table 5:** Analysis of common method bias.

	χ^2^	df	*p*	χ^2^/df	RMSEA	CFI	IFI	TLI
Measurement Model	863.307	450	<0.001	1.918	0.048	0.936	0.937	0.925
Controlled Model	648.737	417	<0.001					
Stepwise χ^2^ Analysis	Δχ^2^	Δdf		Accepted Model				
M.M-C.M	214.57	33	>0.05	Measurement Model				

Note: Define all acronyms in the table—for your consideration.

**Table 6:** Common method bias.

Construct	Item	λ	λ (CMV)	λ-λ (CMV)	SE	CR	α	AVE	C.R
Compassion	Com1	0.718	0.723	0.005	-	-	0.873	0.639	0.881
	Com2	0.647	0.621	0.026	0.071	12.855			
	Com3	0.929	0.936	0.007	0.087	14.407			
Positive Work-related Identity	PWRI1	0.897	0.773	0.124	-	-	0.916	0.834	0.952
	PWRI2	0.890	0.742	0.148	0.038	26.156			
	PWRI3	0.765	0.643	0.122	0.043	19.995			
	PWRI4	0.866	0.789	0.077	0.039	24.802			
Positive Psychological Capital	PsyCapital1	0.661	0.691	0.03	-	-	0.921	0.712	0.947
	PsyCapital2	0.708	0.692	0.016	0.082	12.683			
	PsyCapital3	0.655	0.717	0.062	0.079	11.838			
	PsyCapital4	0.687	0.661	0.026	0.083	12.362			
	PsyCapital5	0.648	0.518	0.13	0.081	11.758			
	PsyCapital6	0.669	0.531	0.138	0.072	12.057			
	PsyCapital7	0.675	0.601	0.074	0.075	12.222			
	PsyCapital8	0.654	0.521	0.133	0.076	11.915			
	PsyCapital9	0.482	0.458	0.024	0.077	9.052			
	PsyCapital10	0.626	0.596	0.03	0.077	11.425			
	PsyCapital11	0.662	0.505	0.157	0.076	12.040			
	PsyCapital12	0.595	0.438	0.157	0.076	10.912			
	PsyCapital13	0.555	0.423	0.132	0.080	10.248			
	PsyCapital15	0.521	0.376	0.145	0.074	9.683			
	PsyCapital16	0.503	0.319	0.184	0.077	9.348			
	PsyCapital18	0.510	0.477	0.033	0.081	9.469			
	PsyCapital20	0.544	0.373	0.171	0.071	10.060			
	PsyCapital21	0.462	0.527	0.065	0.078	8.646			
	PsyCapital22	0.450	0.56	0.11	0.077	8.416			
	PsyCapital23	0.602	0.652	0.05	0.088	11.027			
	PsyCapital24	0.534	0.675	0.141	0.083	9.964			
Job Performance	JP1	0.669	0.676	0.007	-	-	0.880	0.614	0.888
	JP2	0.717	0.718	0.001	0.078	14.148			
	JP3	0.726	0.654	0.072	0.100	12.397			
	JP4	0.706	0.645	0.061	0.098	12.027			
	JP5	0.662	0.626	0.036	0.102	11.536			
χ^2^(417) = 648.737 (*p* = 0.000), CFI = 0.965, TLI = 0.955, IFI = 0.965, RMSEA = 0.037, RMR = 0.021									

Note: Com = compassion; PWRI = positive work-related identity; PsyCapital = positive psychological capital; JP = job performance.

## Data Availability

The data presented in this study are available on request from the corresponding author.

## Appendix A. Survey Items

Compassion

1. I frequently experience compassion on the job.
2. I frequently experience compassion from my supervisor.
3. I frequently experience compassion from my co-workers.

Positive Work-Related Identity

1. As a member of this organization, I am moving closer to who I desire to be.
2. As a member of this organization, I am a virtuous person.
3. As a member of this organization, I am adapting my identity to fit with the organization’s identity.
4. As a member of this organization, I find that different sides of myself fit together.

Positive Psychological Capital

1. I feel confident analyzing a long-term problem to find a solution.
2. I feel confident in representing my work area in meetings with management.
3. I feel confident contributing to discussions about the company’s strategy.
4. I feel confident helping to set targets/goals in my work area.
5. I feel confident contacting people outside the company (e.g., suppliers, customers) to discuss problems.
6. I feel confident presenting information to a group of colleagues.
7. If I should find myself in a jam at work, I could think of many ways to get out of it.
8. At the present time, I am energetically pursuing my work goals.
9. There are lots of ways around any problem.
10. Right now, I see myself as being pretty successful at work.
11. I can think of many ways to reach my current work goals.
12. At this time, I am meeting the work goals that I have set for myself.
13. When I have a setback at work, I have trouble recovering from it, moving on.(R)
14. I usually manage difficulties one way or another at work.
15. I can be “on my own,” so to speak, at work if I have to.
16. I usually take stressful things at work in stride.
17. I can get through difficult times at work because I’ve experienced difficulty before.
18. I feel I can handle many things at a time at this job.
19. When things are uncertain for me at work, I usually expect the best.
20. If something can go wrong for me work-wise, it will.
21. I always look on the bright side of things regarding my job.
22. I’m optimistic about what will happen to me in the future as it pertains to work.
23. In this job, things never work out the way I want them to.
24. I approach this job as if every cloud has a silver lining.

Job Performance

1. This person makes significant contributions to the overall performance of our work unit.
2. This person is one of the best employees in our work unit.
3. This person always completes job assignments on time.
4. This person’s work performance always meets the expectation of the supervisor.
5. This person always actively seeks improvement measures for work enhancement.
